# Supplementary material for: Depressive symptoms and violence exposure in a population-based sample of adult women in South Africa
Source: PLOS Glob Public Health. 2022 Nov 2;2(11):e0001079. doi: 10.1371/journal.pgph.0001079 (PMC10021317; doi:10.1371/journal.pgph.0001079)
Supplement: S1 Appendix — Table A: Mixed effects model of elevated depressive symptoms at cutpoint of PHQ >3 in a stratified cluster sample of women (n = 6093). Table B: Mixed effects model of elevated depressive symptoms assuming non-completers are violence-free (n = 6093). Table C: Mixed effects model of elevated depressive symptoms assuming non-completers are violence-exposed (n = 6093). (DOCX) [file pgph.0001079.s001.docx]

S1 Appendix

| **Table A: Mixed effects model of elevated depressive symptoms at cutpoint of PHQ >3 in a stratified cluster sample of women (*n*=6093)** | | | | | | | |
| --- | --- | --- | --- | --- | --- | --- | --- |
|  | **aOR** | **95% CI** | | | | | **p value** |
| No violence ever | Ref |  |  |  |  |  |  |
| Childhood abuse | 1.24 | ( | 1.06 | - | 1.45 | ) | 0.008 |
| Past-year SIPV exposure | 1.71 |  | 0.83 |  | 3.52 |  | 0.142 |
| Both childhood & SIPV | 1.68 | ( | 1.20 | - | 2.36 | ) | 0.002 |
| Age | 1.01 | ( | 0.99 | - | 1.04 | ) | 0.328 |
| Food secure |  |  |  |  |  |  |  |
| Moderate food insecurity | 1.38 | ( | 1.16 | - | 1.64 | ) | <0.001 |
| Severe food insecurity | 2.03 |  | 1.58 |  | 2.60 |  | <0.001 |
| Social class | 0.92 | ( | 0.89 | - | 0.95 | ) | <0.001 |
|  |  |  |  |  |  |  |  |
| ICC variance by Ward | 0.08 | ( | 0.05 | - | 0.12 | ) | <0.001 |
| aOR: adjusted odds ratio; SIPV: sexual or intimate partner violence; ICC: intra-class correlation | | | | | | | |
| Model accounts for clustering by ward | | | | | | |  |
|  |  |  |  |  |  |  |  |
|  |  |  |  |  |  |  |  |
| **Table B: Mixed effects model of elevated depressive symptoms assuming non-completers are violence-free (*n*=6093)** | | | | | | | |
|  | **aOR** | **95% CI** | | | | | **p value** |
| No violence ever | Ref |  |  |  |  |  |  |
| Childhood abuse | 1.28 | ( | 1.16 | - | 1.42 | ) | <0.001 |
| Past-year SIPV exposure | 1.28 |  | 0.70 |  | 2.33 |  | 0.416 |
| Both childhood & SIPV | 2.00 | ( | 1.52 | - | 2.64 | ) | <0.001 |
| Age | 1.07 | ( | 1.05 | - | 1.08 | ) | <0.001 |
| Food secure |  |  |  |  |  |  |  |
| Moderate food insecurity | 1.30 | ( | 1.15 | - | 1.46 | ) | <0.001 |
| Severe food insecurity | 1.87 |  | 1.55 |  | 2.26 |  | <0.001 |
| Social class (latent) | 0.90 | ( | 0.88 | - | 0.92 | ) | <0.001 |
|  |  |  |  |  |  |  |  |
| ICC variance by Ward | 0.06 | ( | 0.04 | - | 0.08 | ) | <0.001 |
| aOR: adjusted odds ratio; SIPV: sexual or intimate partner violence; ICC: intra-class correlation | | | | | | | |
| Model accounts for clustering by ward | | | | | | |  |
|  |  |  |  |  |  |  |  |
|  |  |  |  |  |  |  |  |
| **Table C: Mixed effects model of elevated depressive symptoms assuming non-completers are violence-exposed (*n*=6093)** | | | | | | | |
|  | **aOR** | **95% CI** | | | | | **p value** |
| No violence ever | Ref |  |  |  |  |  |  |
| Childhood abuse | 1.31 | ( | 1.16 | - | 1.47 | ) | <0.001 |
| Past-year SIPV exposure | 1.28 |  | 0.70 |  | 2.34 |  | 0.418 |
| Both childhood & SIPV | 1.22 | ( | 1.04 | - | 1.42 | ) | 0.012 |
| Age | 1.06 | ( | 1.04 | - | 1.08 | ) | <0.001 |
| Food secure |  |  |  |  |  |  |  |
| Moderate food insecurity | 1.31 | ( | 1.16 | - | 1.47 | ) | <0.001 |
| Severe food insecurity | 1.90 |  | 1.57 |  | 2.30 |  | <0.001 |
| Social class (latent) | 0.90 | ( | 0.88 | - | 0.92 | ) | <0.001 |
|  |  |  |  |  |  |  |  |
| ICC variance by Ward | 0.06 | ( | 0.04 | - | 0.08 | ) | <0.001 |
| aOR: adjusted odds ratio; SIPV: sexual or intimate partner violence; ICC: intra-class correlation | | | | | | | |
| Model accounts for clustering by ward | | | | | | |  |
