# Supplementary material for: Depressive symptoms and violence exposure in a population-based sample of adult women in South Africa
Source: PLOS Glob Public Health. 2022 Nov 2;2(11):e0001079. doi: 10.1371/journal.pgph.0001079 (PMC10021317; doi:10.1371/journal.pgph.0001079)
Supplement: Attachment — Submitted filename: Hatcher Rebuttal 14Jun22.docx [file pgph.0001079.s002.docx]

Dear Dr. Waqas and PLoS Global Health Reviewers,

We have responded to editor and reviewer comments and appreciate your consideration of this revision.

Warm regards,

Authors

Journal Requirements:

1. Please send a completed 'Competing Interests' statement, including any COIs declared by your co-authors. If you have no competing interests to declare, please state "The authors have declared that no competing interests exist".

*We have completed the Competing Interests statement.*

2. Please amend your detailed Financial Disclosure statement. This is published with the article. It must therefore be completed in full sentences and contain the exact wording you wish to be published.

a. State the initials, alongside each funding source, of each author to receive each grant.
b. State what role the funders took in the study. If the funders had no role in your study, please state: “The funders had no role in study design, data collection and analysis, decision to publish, or preparation of the manuscript.”
c. If any authors received a salary from any of your funders, please state which authors and which funders.

*Financial Disclosure statement has now been amended.*

3. We notice that your supplementary tables are included in the manuscript file. Please remove them and upload them with the file type 'Supporting Information'. Please ensure that each Supporting Information file has a legend listed in the manuscript after the references list.

*Thanks we have now done this.*

4. Please note that your Data Availability Statement is currently missing a direct link to access each database. If your manuscript is accepted for publication, you will be asked to provide these details on a very short timeline. We therefore suggest that you provide this information now, though we will not hold up the peer review process if you are unable.

*We are able to include this now:*

**Data Availability Statement**

Public access data for use under a Creative Commons CC-BY-SA (Attribution plus Share-Alike) Licens is available at <https://www.datafirst.uct.ac.za/dataportal/index.php/catalog/874> or by requesting access to **support@data1st.org.**

Reviewer #1: I want to appreciate the authors for their time and effort in doing this very important public health issue. The following comment needs to be addressed for the additional value of the paper. The paper needs to be revised the grammar, editing (you used present illness), list acronyms/ abbreviations (when you used in the first line for example line 110, PHQ), and avoid using words such as our, us, and we throughout the paper.

*Thanks we have edited, ensured the spelling out of acronyms in the first instance, and removed first-person or second-person language.*

1. The Abstract section is good but the conclusion is too general, in this you also said that "Individual markers of poverty" and "Reduction of poverty" what do you mean. Your conclusion is general it should be specific and based on your finding.

*We added precision to this statement in the Abstract:*

Exposure to violence in childhood or past-year SIPV were associated with depressive symptoms among women. These associations persist after controlling for socio-economic markers and latent neighborhood characteristics. These data suggest that efforts to reduce the burden of depressive symptoms may benefit from approaches that prevent violence against women and children.

3. In the Introduction section, Paragraph two (line 62) the way of citation need to be corrected and what does elsewhere mean?

*We omitted the term “elsewhere” and fixed the citations. Thanks for this.*

4. In general, the introduction is good, but you failed to address, depression and violence from global to local and what efforts was done in South Africa, consequence, magnitude, and severity of violence and depression are not clearly addressed in this section.

*That is a good point. We have now added two paragraphs to characterize the burden of disease in terms of depression and violence:*

The Global Burden of Disease Study found that depressive disorders are among the most important causes of years lost to disability, especially among women,^[2]^ and this trend has only heightened since the start of the SARS-Cov2 pandemic.^[3]^ In South Africa, national surveys suggest probable depression occurs among up to 28% of women,^[4]^ with considerable variation across the country.^[5]^

Depressive symptoms among women in South Africa may be associated with their recent or childhood exposure to violence. Intimate partner violence (physical and/or sexual abuse by a current or ex-boyfriend^[6]^) is high in South Africa, with one population-based survey noting past-year exposure rates to sexual (8%) or physical (13%) violence.^[7]^ Similarly sexual violence in the form of rape by a partner or non-partner is higher in South Africa than most settings globally, with 25-28% reporting ever experiencing rape.^[7, 8]^ Childhood violence us similarly high in South Africa, with exposure to physical or sexual violence reported by 15% of girls in South Africa national population-based study.^[9]^

5. In the method section, it is good but there is no clear information about the study area, study time, study design, population (source and study population/inclusion and exclusion criteria), sample size determination, sampling technique, and data quality control separately (subtitle) in this paper.

*We have bolstered this section with the sub-headings requested:*

***Study population and sampling***

Quality of Life survey 6 interviewed 13 616 adults across Gauteng, South Africa between the time of Sept 2019 – August 2020. The multistage stratified cluster sample design covered urban, peri-urban, and rural areas within the province and has been described fully elsewhere.^[26]^ South African local government assigns neighborhoods as administrative wards. Within each (total of *n*=529 wards), 5 to 6 Enumeration Areas (EAs) were sampled on a probability proportional to size (PPS) basis. PPS uses population data to assign a sampling probability variable so that between 4 to 6 dwelling units were randomly selected within each EA. A floor of 20 interviews was conducted in each ward. Data was weighted by population size, race and gender to ensure representative estimates.

***Procedures***

On arrival at each dwelling unit, a trained enumerator spoke with residents to list all adults living in the dwelling. In-field random sampling (using a tablet) selected one resident adult from a household roster for interview. Participants were eligible if they were 18 years or older, willing to take part, and cognitively able to complete the interview.

Interviews were conducted in-person by trained enumerators following verbal informed consent. Participants were given a printed information sheet to keep, if they chose. All study materials were translated in nine local languages, and interviews were conducted in the language of choice.

***Quality control***

As described fully elsewhere,^[33]^ data was quality was checked by quality assurance managers at the field level on a daily basis. Built-in checks were implemented through queries built into real-time data entry and in a back-end audit trail. Call backs were implemented to 26.6% of the full sample to check basic socio-demographics against the dataset. Any records that did not pass each of these three quality checks were omitted from the final dataset.

6. The measurement (operational definitions) is good but the way that you measured some variables is somehow confused. Line 119 you said that "These 4 items have not been validated", in this, the definition of an intimate partner is defined as also for non-partner (why?), how did you measure this?

*This is a good point that deserves distinction in the Methods section. We added:*

The rationale for including this broader definition of SIPV is that rape in South Africa (by a partner or non-partner) is considered a pressing health and policy issue. Therefore, the survey wanted to establish baseline levels of exposure to both partner perpetration and non-partner perpetration.

7. Also, intimate partner violence is broad and includes physical, sexual, and psychological violence but you only used some of them?

*We only use some of the items based on Item Response Theory and the brief scope of this additional module to the overall survey.*

8. The socioeconomic status and food insecurity measurement are also not clear (why you did not use Principal component analysis for the economic status?)

*This is a good point and we have added it to the Limitations:*

The non-validated measure of food insecurity and the continuous additive scale for social class has limitations compared to existing measures or principal component analysis, but these poverty markers needed to align with past survey rounds in order to track trends over time.

9. In the ethical considerations, you included the funding issue, it is better to list in the funding report.

*Agreed, we have now pulled this into a Funding Statement.*

10. In the Result section, avoid words that are not scientific such as Just (line 161).

*Thanks we have edited this section.*

11. Avoid (columns 2-4) in line 167 when you referred to table 1. It would be better for readers if you show the results of depressed symptoms and violence in a separate paragraph (make in one paragraph from lines 170-179) b/ce it is about violence.

*Thanks this suggestion does make the presentation clearer.*

12. Line 183, change the word "lacking high school education"

*We changed this to “no high school education.”*

13. Discussion: In this, some parts of the justification are cited, it be better to justify the possible reasons for both the magnitudes and for factors (for example lines 227-236).

*We improved this paragraph through additional citations:*

We found that latent characteristics of one’s ward of residence seem to explain considerable variation in depressive symptoms, over and above individual social class and food security. There seem to be multiple plausible reasons for neighborhood residence and depressive symptoms. The physical infrastructure of a community can be linked to depressive symptoms by causing increased daily stressors and strain.^[22, 36]^ Crime and witnessing violence can directly impact on mental health.^[23, 37]^ It is also possible that those who live in poor socio-economic areas have fewer social or institutional resources to cope with strains or threats to safety when exposed.^[19, 24]^

14. Line 242 what does theoretically mean? The conclusion did not show the finding and not concluded based on your pertinent findings in addition to that you cited the reference (42) while it is unnecessary. Conclude your finding based on your objective.

*We agree and have omitted this statement from the Discussion.*

15. There are no abbreviation, author contribution, funding information, ethical issue, data availability, Acknowledgement, consent and conflict of interest statement in this paper. Follow the guideline of the journal for tables and edit some of the references and avoid reference number 42 from the citation (conclusion) and reference list.

*Thanks we have now included each of these sections.*

Reviewer #2: Overall, this is a well-written manuscript. The authors have systematically examined the topic of investigation.
There are a few specific comments as mentioned below.
1. Line 34-38: Abstract: - Mention Odds Ratio and 95% confidence interval for significant associations.

*Thanks for this, we have now included those:*

Childhood abuse alone increases the odds of high depressive symptomology, after controlling for individual-level markers of poverty and neighborhood of residence (aOR 1.31, 95%, CI 1.17-1.37). Combined exposure to childhood abuse and past-year SIPV increased odds of reporting elevated depressive symptoms (aOR 2.05, 95%, CI 1.54-2.71).

2. Line 82-86: Elaborate on sampling method used. How many total wards and Enumeration Areas are there in the province? Do they cover rural, urban or both areas? What proportion was chosen for PPS sampling? Which type of random sampling was used to select dwelling units? were chosen out of how many total wards available? If more than one adult women were there in a dwelling, how were they selected?

*We bolstered this section:*

***Study population and sampling***

Quality of Life survey 6 interviewed 13 616 adults across Gauteng, South Africa between the time of Sept 2019 – August 2020. The multistage stratified cluster sample design covered urban, peri-urban, and rural areas within the province and has been described fully elsewhere.^[26]^ South African local government assigns neighborhoods as administrative wards. Within each (total of *n*=529 wards), 5 to 6 Enumeration Areas (EAs) were sampled on a probability proportional to size (PPS) basis. PPS uses population data to assign a sampling probability variable so that between 4 to 6 dwelling units were randomly selected within each EA. A floor of 20 interviews was conducted in each ward. Data was weighted by population size, race and gender to ensure representative estimates.

***Procedures***

On arrival at each dwelling unit, a trained enumerator spoke with residents to list all adults living in the dwelling. In-field random sampling (using a tablet) selected one resident adult from a household roster for interview. Participants were eligible if they were 18 years or older, willing to take part, and cognitively able to complete the interview.

3. Line 106-107, 114-115, 120-121: Were the data collection tools translated to local language? Mention it clearly.

*Thanks we clarified this:*

All study materials were translated in nine local languages, and interviews were conducted in the language of choice.

4. Line 138-139: Description of a ward should be part of methods section where the study area is mentioned. Move it there.

*Good point, it is now included there:*

South African local government assigns neighborhoods as administrative wards. Within each (total of *n*=529 wards), 5 to 6 Enumeration Areas (EAs) were sampled on a probability proportional to size (PPS) basis.

5. Line 188-192: Provide Odds Ratio and 95% confidence interval in bracket for significant associations.

*Thanks we now provide all relevant estimates and confidence intervals:*

Compared to women reporting no violence, those with childhood violence had 31% higher odds of adult depressive symptoms (aOR 1.31, 95%, CI 1.17-1.37). The small number of women reporting past-year SIPV but never reporting childhood abuse also had higher odds of depressive symptoms, but this was not statistically significant (aOR 1.31, 95%, CI 0.72-2.39). Those reporting both childhood abuse and past-year SIPV had more than doubled odds of high depressive symptoms (aOR 2.05, 95%, CI 1.54-2.71).

While revising your submission, please upload your figure files to the Preflight Analysis and Conversion Engine (PACE) digital diagnostic tool, <https://pacev2.apexcovantage.com/>. PACE helps ensure that figures meet PLOS requirements. To use PACE, you must first register as a user. Registration is free. Then, login and navigate to the UPLOAD tab, where you will find detailed instructions on how to use the tool. If you encounter any issues or have any questions when using PACE, please email PLOS at [figures@plos.org](mailto:figures@plos.org). Please note that Supporting Information files do not need this step.
