# Supplementary material for: Depressive symptoms and violence exposure in a population-based sample of adult women in South Africa
Source: PLOS Glob Public Health. 2022 Nov 2;2(11):e0001079. doi: 10.1371/journal.pgph.0001079 (PMC10021317; doi:10.1371/journal.pgph.0001079)
Supplement: Attachment — Submitted filename: Rebuttal.docx [file pgph.0001079.s003.docx]

1. In the Funding Information you indicated that no funding was received. Please revise the Funding Information field to reflect funding received.

Please ensure that the funders and grant numbers match between the Financial Disclosure field and the Funding Information tab in your submission form. Note that the funders must be provided in the same order in both places as well.

*Thanks we have ensured alignment.*

2. We have noticed that you have uploaded Supporting Information files, but you have not included a list of legends. Please add a full list of legends for your Supporting Information files after the references list.

*A full list of legends has been included at bottom of manuscript.*

3. Please review your reference list to ensure that it is complete and correct. If you have cited papers that have been retracted, please include the rationale for doing so in the manuscript text, or remove these references and replace them with relevant current references. Any changes to the reference list should be mentioned in the rebuttal letter that accompanies your revised manuscript. If you need to cite a retracted article, indicate the article’s retracted status in the References list and also include a citation and full reference for the retraction notice.

*We have reviewed the full reference list and it is correct.*

4. Have the authors made all data underlying the findings in their manuscript fully available (please refer to the Data Availability Statement at the start of the manuscript PDF file)?

The [PLOS Data policy](https://journals.plos.org/globalpublichealth/s/data-availability) requires authors to make all data underlying the findings described in their manuscript fully available without restriction, with rare exception. The data should be provided as part of the manuscript or its supporting information, or deposited to a public repository. For example, in addition to summary statistics, the data points behind means, medians and variance measures should be available. If there are restrictions on publicly sharing data—e.g. participant privacy or use of data from a third party—those must be specified.

Reviewer #1: Yes

Reviewer #2: No

*The Data Availability Statement shows that the public repository is accessible:*

“Public access data for use under a Creative Commons CC-BY-SA (Attribution plus Share-Alike) License is available at <https://www.datafirst.uct.ac.za/dataportal/index.php/catalog/874> or by requesting access to **support@data1st.org.”**

Reviewer #1: Majority of the comments are addressed. But there are some editorial errors that still need to be addressed throughout the paper. Subjective words such as our, us are also still common in the paper that needs to be corrected.

*We have omitted the words “our”, “us”, and “we”.*

The conclusion part is still not corrected. No need of citation in the conclusion part and you should conclude based on your pertinent finding and avoid citation.

*We have removed the citation in Conclusion and reframed:*

*“*Exposure to violence was independently associated with high depressive symptoms among a relatively large, population-based sample of women in South Africa. These data may contribute to national efforts to reduce violence against women and girls. Global efforts to improve mental health could gain traction by supporting individuals and communities who are exposed to high rates of violence. Preventing violence represents a crucial step for ensuring health and well-being for women and children globally. “

Reviewer #2: There is a good improvement in the revised version.
1. There is still a need to make the sampling description more clear and specific. What proportion was chosen for PPS sampling? Which type of random sampling was used to select dwelling units? How was the total sample size reached at by combining respondents selected at all levels? If more than one adult respondents were available in a dwelling, how were they selected?

*We answered each of these questions in the Procedures section:*

***“Study population and sampling***

Quality of Life survey 6 interviewed 13 616 male and female adults across Gauteng, South Africa between the time of Sept 2019 – August 2020. The multistage stratified cluster sample design covered urban, peri-urban, and rural areas within the province and has been described fully elsewhere.^[26]^ South African local government assigns neighborhoods as administrative wards. Within each (total of *n*=529 wards), 5 to 6 enumeration areas were sampled on a probability proportional to size (PPS) basis. PPS uses population data to assign a sampling probability variable so that between 4 to 6 dwelling units were randomly selected within each enumeration areas, using a randomly generated interval. A fixed sample size was set for each ward in the province (dependent on municipal location). Within the ward, each numeration area was assigned a sampling weight according to the number of dwelling unit, meaning the probability of selecting an enumeration area was proportional to its relative size in terms of number of dwelling units within the ward. A floor of 20 interviews was conducted in each ward. Data was weighted by population size, race and gender to ensure representative estimates.

***Procedures***

On arrival at each dwelling unit, a trained enumerator spoke with residents to list all adults living in the dwelling. In-field random sampling selected one resident adult from a household roster for interview. This was achieved in practice by manually listing all resident adults and programming the data collection system (on tablet computers) to randomly select one adult to be interviewed. Participants were eligible if they were 18 years or older, willing to take part, and cognitively able to complete the interview.”

2. In Line 113, it is mentioned that 13616 interviews were carried out. But, the same number is regarded as the overall study population in Line 219. It also states that 7276 women were included as the sample. Were there a total of 7276 women in the database of 13616 respondents which was used as part of this study? If they were more, how were these 7276 women chosen? There is a need to present these details with more clarity.

*We clarified that all adult females were included in this analysis:*

*“*Quality of Life survey 6 interviewed 13 616 male and female adults across Gauteng, South Africa between the time of Sept 2019 – August 2020.”

*“*A total of 7 276 adult females were included in the sample for this analysis.”
